# Complement activity is regulated in C3 glomerulopathy by IgG–factor H fusion proteins with and without properdin targeting domains

**DOI:** 10.1016/j.kint.2020.09.028

**Published:** 2021-02

**Authors:** Alyssa C. Gilmore, Yuchun Zhang, H. Terence Cook, Deborah P. Lavin, Suresh Katti, Yi Wang, Krista K. Johnson, SungKwon Kim, Matthew C. Pickering

**Affiliations:** 1 Centre for Inflammatory Disease, Imperial College London, UK; 2 Alexion Pharmaceuticals, New Haven, Connecticut, USA

**Keywords:** complement, glomerulonephritis, immunology

## Abstract

C3 glomerulopathy is characterized by accumulation of complement C3 within glomeruli. Causes include, but are not limited to, abnormalities in factor H, the major negative regulator of the complement alternative pathway. Factor H-deficient (*Cfh*^-/-^) mice develop C3 glomerulopathy together with a reduction in plasma C3 levels. Using this model, we assessed the efficacy of two fusion proteins containing the factor H alternative pathway regulatory domains (FH_1-5_) linked to either a non-targeting mouse immunoglobulin (IgG-FH_1-5_) or to an anti-mouse properdin antibody (Anti-P-FH_1-5_). Both proteins increased plasma C3 and reduced glomerular C3 deposition to an equivalent extent, suggesting that properdin-targeting was not required for FH_1-5_ to alter C3 activation in either plasma or glomeruli. Following IgG-FH_1-5_ administration, plasma C3 levels temporally correlated with changes in factor B levels whereas plasma C5 levels correlated with changes in plasma properdin levels. Notably, the increases in plasma C5 and properdin levels persisted for longer than the increases in C3 and factor B. In *Cfh*^-/-^ mice IgG-FH_1-5_ reduced kidney injury during accelerated serum nephrotoxic nephritis. Thus, our data demonstrate that IgG-FH_1-5_ restored circulating alternative pathway activity and reduced glomerular C3 deposition in *Cfh*^-/-^ mice and that plasma properdin levels are a sensitive marker of C5 convertase activity in factor H deficiency. The immunoglobulin conjugated FH_1-5_ protein, through its comparatively long plasma half-life, may be a potential therapy for C3 glomerulopathy.

## Graphical abstract

**Figure:**
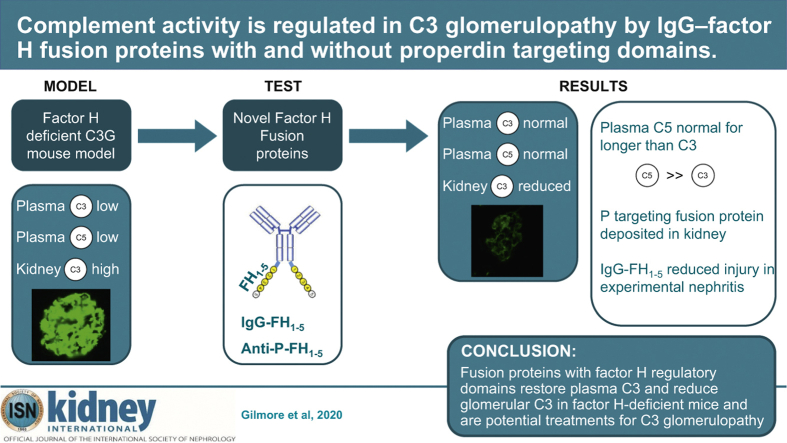

**Translational Statement**

C3 glomerulopathy (C3G) is a kidney disease characterized by abnormal accumulation of complement C3 within glomeruli and glomerular damage. It is due to uncontrolled activation of the complement alternative pathway. Fusion proteins, comprised of functional domains of the alternative pathway regulator, factor H (FH), linked to an antibody, restored complement regulation, and reduced glomerular C3 in a C3G mouse model. The non-targeting antibody conjugation resulted in a comparatively long fusion protein plasma half-life, and this, or similar conjugation methods, could be used to augment FH function in the therapy of C3G.

C3 glomerulopathy (C3G) is a complement-mediated renal disorder characterized by abnormal amounts of C3 within glomeruli.^1^ It can progress to renal failure and has no definitive treatment.^2^ C3G is associated with abnormal activation of the complement alternative pathway (AP). AP activation results in the production of C3bBb (C3 convertase), an enzyme that cleaves C3. After the addition of another C3b molecule, the resulting C3bBbC3b complex (C5 convertase) can cleave complement C5. Uncontrolled AP activation in C3G can be associated with reduction in circulating C3, C5, factor B (FB), and properdin. Causes of uncontrolled AP activation include loss of function changes in complement regulators and gain of function changes in complement activators.^2^ The key negative AP regulator is factor H (FH), and FH deficiency in humans, pigs, and mice is associated with C3G.^3^ C3 nephritic factor, an antibody that stabilizes the AP C3 convertase, is frequent in C3G.^4^ C3 nephritic factor may result in reduction in C3 alone (properdin-independent C3 nephritic factor) or reduction in both C3 and C5 (properdin-dependent C3 nephritic factor).^5^^,^^6^

Restoring complement regulation should ameliorate C3G because C3 dysregulation is the central defect in pathogenesis. Accordingly, FH-deficient mice (*Cfh*^–/–^) did not develop spontaneous C3G if the mice were also deficient in FB.^7^ Properdin is a positive regulator of the AP C3 convertase, so its removal would be predicted to reduce C3 and C5 activation. In the *Cfh*^–/–^ mouse strain, properdin deficiency unexpectedly exacerbated glomerular C3 deposition.^8^^,^^9^ Although properdin deficiency did not ameliorate plasma C3 levels, C5 levels increased, suggesting that properdin was necessary for the activity of the C5 but not C3 convertase.^8^^,^^9^

Administration of mouse^10^ or human FH^11^^,^^12^ to *Cfh*^–/–^ mice reduced glomerular C3 staining and increased circulating C3 levels. Constructs that contain only the regulatory and targeting domains of FH (mini-FH molecules) are also efficacious in this model.^13^ Other approaches include proteins that target sites of complement activation. These include TT30,^14^ a protein containing the complement regulatory domains of FH (FH_1-5_) linked to the complement-binding domains of complement receptor 2, and homodimeric FH molecules,^15^ mini-FH molecules that contain complement-binding domains of factor H–related protein 1.

Because the therapeutic utility of inhibiting complement C3 activation is under investigation in C3G,^2^ it is important to understand the kinetics of glomerular C3 deposition and its relationship to AP activation within both glomeruli and the circulation. In models of immune complex–mediated glomerulonephritis, glomerular C3c cleared within 24 hours of preventing complement activation, whereas glomerular C3d persisted for weeks.^16^ Similarly, in *Cfh*^–/–^ mice, exogenous FH results in reduction in glomerular C3c staining at 24 hours, whereas glomerular C3d remains unchanged.^11^

In this study, we investigated the efficacy of 2 novel fusion proteins with complement regulatory activity in the *Cfh*^–/–^ mouse model of C3G. The proteins contained the regulatory domains of mouse FH (FH_1-5_), which was conjugated to mouse Ig to prolong the biological half-life. One of the fusion proteins, termed IgG-FH_1-5_, was conjugated to a non-targeting mouse monoclonal antibody. The other, termed anti-P-FH_1-5_, was conjugated to a monoclonal antibody to mouse properdin. This was done to test the hypothesis that targeting this fusion protein to sites of complement activation, by interacting with properdin deposition, would enhance tissue complement regulation. Our data demonstrate that both the non-targeting (IgG-FH_1-5_) and the properdin-targeting (anti-P-FH_1-5_) proteins restored plasma and glomerular C3 regulation in *Cfh*^–/–^ mice. Time course studies showed that restoration of C3 and FB levels mirrored levels of the fusion proteins in the circulation. In contrast, increases in plasma C5 persisted after the fusion proteins had cleared from the circulation. We show that IgG-FH_1-5_ ameliorated glomerular injury during accelerated serum nephrotoxic nephritis in *Cfh*^–/–^ mice.

## Results

### Generation of fusion proteins and *in vitro* and *in vivo* assessment of activity

**Figure 1:**
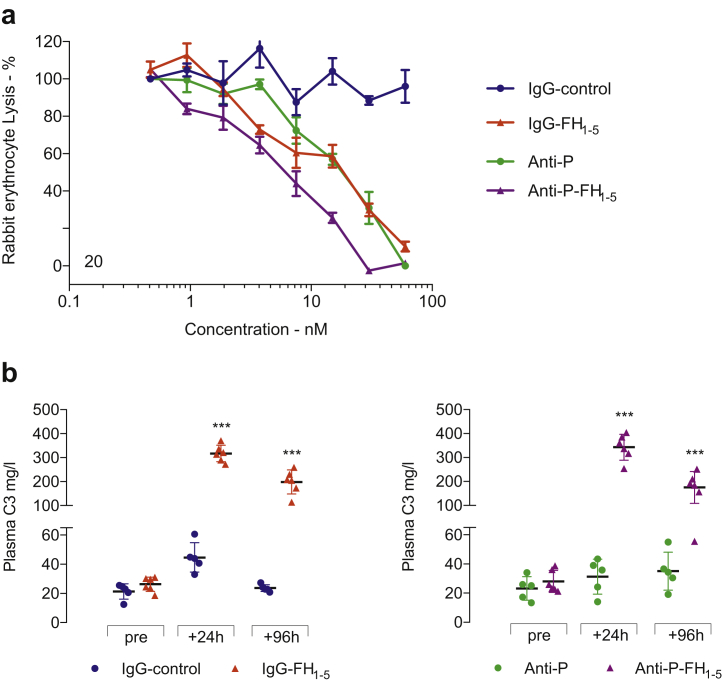
**(a) Complement alternative pathway–dependent hemolysis assay.** Antibodies were titrated in 2-fold dilutions from 60 nM through to 0.5 nM. The IgG-FH_1-5_, anti-P-FH_1-5_, and anti-P reagents reduced rabbit erythrocyte hemolysis in a dose-dependent manner. No inhibition was evident using the IgG-control protein. Horizontal bars denote mean values, and error bars represent SD. (**b**) Plasma complement C3 in *Cfh*^–/–^ mice after injection of fusion proteins. Plasma C3 was measured before and 24 hours and 96 hours after administration of IgG-FH_1-5_ (red triangles, n = 5) Anti–P–FH_1-5_ (purple triangles, n = 6), Anti-P (green dots, n = 5), and IgG-control (blue dots, n = 5). Horizontal bars denote mean values, and whiskers denote SD. ∗∗∗*P* ≤ 0.001 versus pretreatment value and derived from 2-way ANOVA with Bonferroni multiple comparisons test. FH, factor H; P, properdin.

Fusion proteins were created by linking the first 5 short consensus repeat (SCR) domains of mouse FH (FH_1-5_) to either an anti-mouse properdin antibody (anti-P-FH_1-5_) or an antibody with a non-targeting Ig domain (IgG-FH_1-5_, Supplementary Figure S1). The activity of the proteins was assessed using an AP-specific hemolytic assay (Figure 1a). Anti-P-FH_1-5_ and IgG-FH_1-5_ inhibited hemolysis in a dose-dependent manner with greater potency for anti-P-FH_1-5_. Anti-properdin (anti-P) also reduced hemolysis in a dose-dependent manner, but the IgG-control had no effect. After equimolar injections of the proteins into *Cfh*^–/–^ mice, the IgG-FH_1-5_ protein was detectable up to 11 days after injection, whereas anti-P-FH_1-5_ was detectable up to 4 days after injection (Supplementary Figure S2). To determine whether IgG-FH_1-5_ and anti-P-FH_1-5_ could restore plasma AP regulation in *Cfh*^–/–^ mice, we first measured plasma C3 levels after injection of the fusion proteins (Figure 1b). Administration of either IgG-FH_1-5_ or anti-P-FH_1-5_ significantly increased plasma C3 at 24 hours and, to a lesser extent, at 96 hours after injection (Figure 1b). These data demonstrated that both IgG-FH_1-5_ and anti-P-FH_1-5_ could temporarily restore plasma AP regulation in *Cfh*^–/–^ mice. We next performed a detailed analysis of these changes by characterizing the time course of changes in not only plasma C3 but also of plasma C5 and the alternative pathway proteins FB and properdin.

### Increases in circulating C5 and properdin levels persist longer than increases in C3 and FB after administration of IgG-FH_1-5_ and anti-P-FH_1-5_ in *Cfh*^–/–^ mice

**Figure 2:**
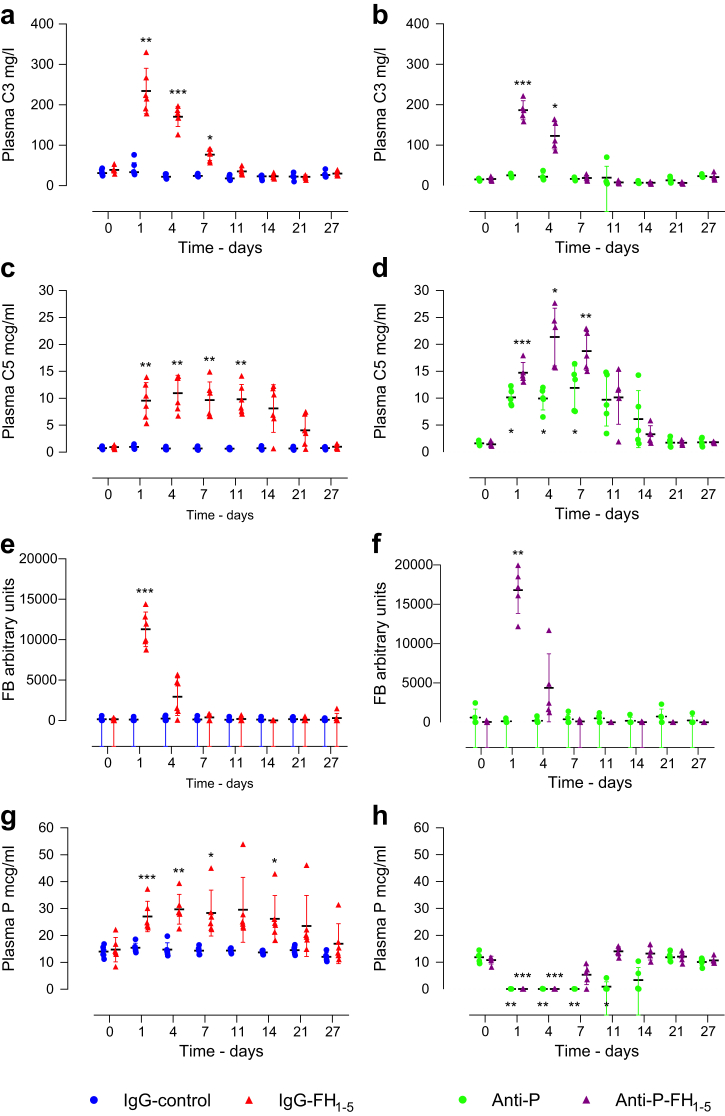
**Plasma complement profile time course in *Cfh***^**–/–**^**mice injected with IgG-FH**_**1-5**_**and anti-P-FH**_**1-5**_**.** Plasma C3 (**a,b**), C5 (**c,d**), FB (**e,f**), and properdin (P; **g,h**) from experimental start until 27 days after single injection of IgG-FH_1-5_ (**a,c,e,g;** red triangles, n = 5) or anti-P-FH_1-5_ (**b,d,f,h**; urple triangles, n = 6). Controls included anti-P (green dots, n = 5) and an isotype-matched monoclonal antibody (IgG-control; blue dots, n = 5). Horizontal bars denote mean values, and whiskers denote SD. ∗*P* ≤ 0.05, ∗∗*P* ≤ 0.01, ∗∗∗*P* ≤ 0.001 versus pretreatment value and derived from 2-way analysis of variance with Bonferroni multiple comparisons test. FB, factor B; FH, factor H.

We compared the kinetics of changes in C3, C5, FB, and properdin by measuring proteins at intervals up to 27 days after a single injection of either IgG-FH_1-5_ or anti-P-FH_1-5_ (Figure 2). After administration of IgG-FH_1-5_, the peak rise in C3, C5, FB, and properdin all occurred at day 1 post-injection. The time for these changes to fall to pretreatment levels differed. The return to pretreatment levels occurred between days 1 and 4 for FB, between days 7 and 11 for C3, and between days 14 and 21 for both properdin and C5 (Figure 2a, c, e, and g). After administration of anti-P-FH_1-5_, peak C3 levels occurred on day 1 but fell to pretreatment levels between days 4 and 7 (Figure 2b). Peak C5 levels occurred on day 4 and returned to pretreatment levels between days 7 and 11 days (Figure 2d). The peak rise in FB occurred on day 1 and fell to pretreatment levels between days 1 and 4 (Figure 2f). As expected, free plasma properdin was depleted after injection of either anti-P-FH_1-5_ or anti-P (Figure 2h). Free plasma properdin levels remained low for up to 7 days after anti-P-FH_1-5_ and up to 14 days after anti-P (Figure 2h). Although both fusion proteins temporarily restored plasma AP regulation, the duration of improvement in C3, FB, and C5 levels reflected the duration of their detection in plasma, which was shorter for anti-P-FH_1-5_ (Supplementary Figure S2). In summary, changes in the levels of C3 and FB after injection of either protein were shorter than those of C5 and properdin. These data suggest that plasma FB is a sensitive marker of C3 convertase activity in this setting, whereas properdin is a marker of C5 convertase activity. Notably, after anti-P injection, there was a rise in C5 at 24 and 96 hours and day 7 (Figure 2d). As reported,^8^^,^^9^ this indicates that the C5 convertase is dependent on properdin in *Cfh*^–/–^ mice.

### IgG-FH_1-5_ and anti-P-FH_1-5_ reduced glomerular C3c but did not alter C3d staining in *Cfh*^–/–^ mice

**Figure 3:**
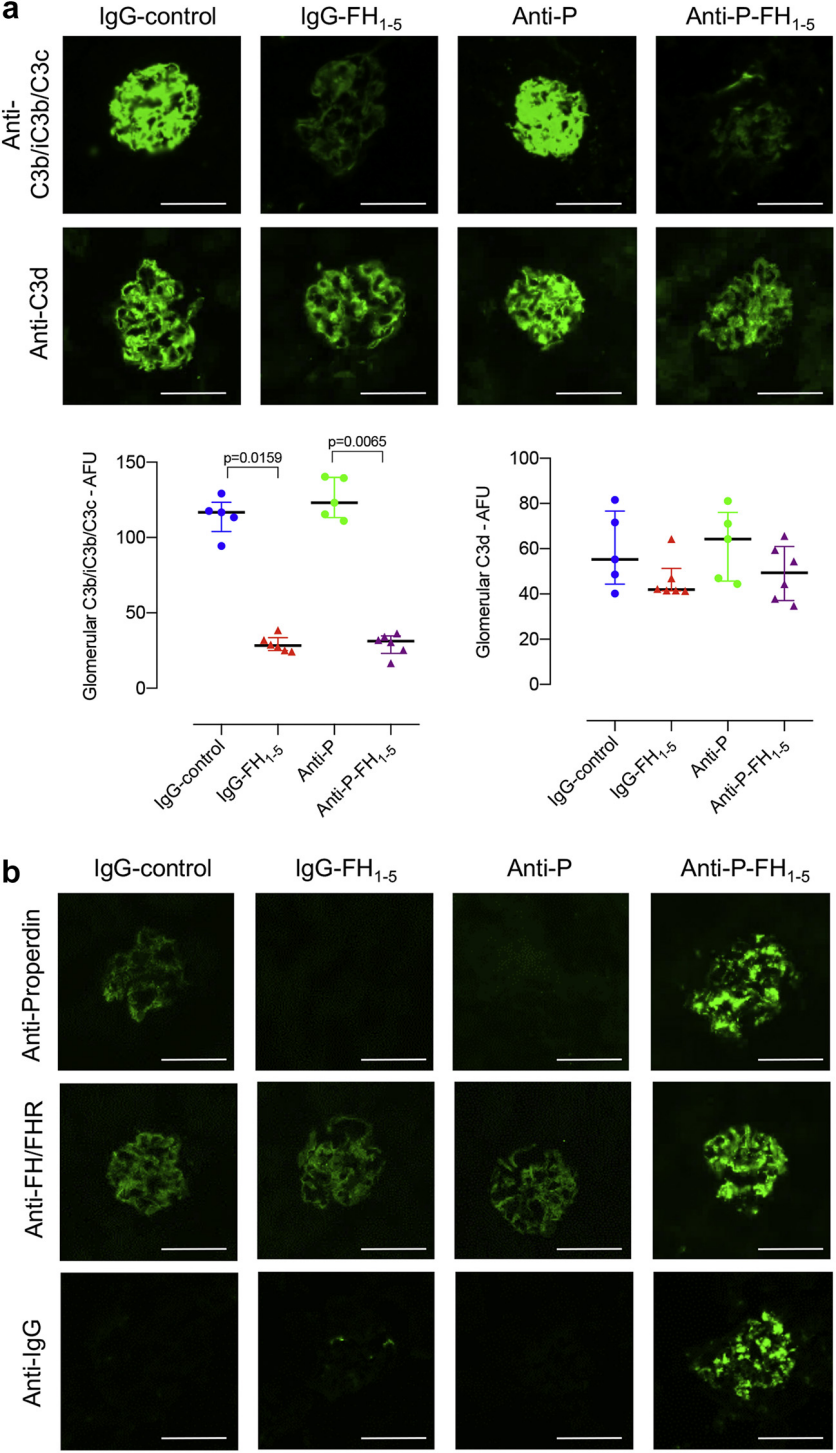
**Glomerular complement immunostaining in *Cfh***^**–/–**^**mice 96 hours after injection of either IgG-FH**_**1-5**_**or anti-P-FH**_**1-5**_**.** (**a**) Representative images together with quantification of glomerular C3b/iC3b/C3c and C3d in the 4 experimental groups: IgG-FH_1-5_ (red triangles, n = 5), anti-P-FH_1-5_ (purple triangles, n = 6), anti-P (green dots, n = 5), and IgG-control (blue dots, n = 5). Plasma C3 levels at the time of cull are shown in Figure 1. (**b**) Data points represent median values, and whiskers denote interquartile range. *P* values derived from Kruskal-Wallis test with Dunn multiple comparisons test. (**b**) Representative images of glomerular IgG, FHR, and P, staining in the 4 experimental groups. Bar = 100 μm. AFU, arbitrary fluorescent units; FH, factor H; P, properdin. To optimize viewing of this image, please see the online version of this article at www.kidney-international.org.

We next assessed the influence of IgG-FH_1-5_ and anti-P-FH_1-5_ on glomerular C3b/iC3b/C3c, C3d, properdin, and FH-related (FHR) proteins. At 96 hours after injection of IgG-FH_1-5_, glomerular C3b/iC3b/C3c staining was reduced but glomerular C3d (Figure 3a) and FHR staining (Figure 3b) was not. Glomerular C3b/iC3b/C3c remained unchanged with either IgG-control or anti-P. The abnormal linear pattern of glomerular properdin staining in untreated *Cfh*^–/–^ mice^9^ remained 96 hours after IgG-control. In *Cfh*^–/–^ mice injected with either anti-P or IgG-FH_1-5_, the glomerular properdin level was almost undetectable at 96 hours (Figure 3b). Unexpectedly, anti-P-FH_1-5_ injection resulted in a granular glomerular staining pattern using anti-properdin, anti-FH/FHR, and anti-IgG antibodies (Figure 3b). This finding suggested that the anti-P-FH_1-5_ protein was being deposited in glomeruli. Injection of anti-P-FH_1-5_ protein into wild-type mice resulted in glomerular staining with anti-properdin, anti-FH/FHR, and anti-IgG antibodies at 96 hours (Supplementary Figure S3), indicating that this reagent interacts with normal glomeruli. However, we speculated that the anti-P-FH_1-5_ protein could also interact with the pre-existing glomerular properdin in *Cfh*^–/–^ mice. When we injected the reagent into *Cfh*^–/–^ mice that had been pretreated with anti-P to remove glomerular properdin, there was a reduction in the granular staining patterns using anti-properdin, anti-FH/FHR, and anti-IgG antibodies (Supplementary Figure S4). These observations indicate that the glomerular interaction of anti-P-FH_1-5_ protein in *Cfh*^–/–^ mice is partly dependent on glomerular properdin.

### IgG-FH_1-5_ ameliorated renal injury during accelerated serum nephrotoxic nephritis in *Cfh*^–/–^ mice

**Figure 4:**
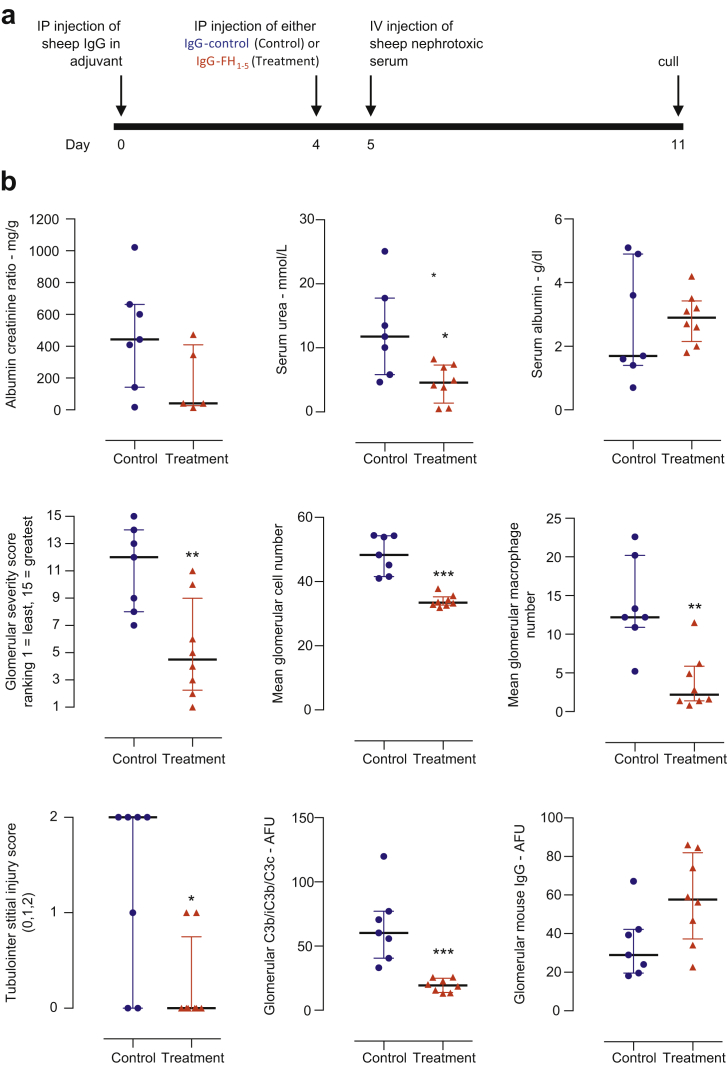
**Pretreatment with IgG-FH**_**1-5**_**ameliorated renal injury during accelerated serum nephrotoxic nephritis in *Cfh***^**–/–**^**mice**. (**a**) Schematic of the accelerated serum nephrotoxic nephritis protocol. Mice pre-immunized with sheep IgG received either IgG-FH_1-5_ (treatment group, n = 8) or IgG-control (control group, n = 7) 24 hours before administration of the sheep nephrotoxic serum. (**b**) Renal function and histology 6 days after induction of accelerated serum nephrotoxic nephritis in mice treated with either IgG-FH_1-5_ (treatment; red triangles, n = 8) or IgG-control (control; blue circles, n = 7). Horizontal bars denote median values, and whiskers denote interquartile range. ∗*P* ≤ 0.05, ∗∗*P* ≤ 0.01, ∗∗∗*P* ≤ 0.001 versus control and derived from Mann-Whitney test. FH, factor H; P, properdin.

In patients with C3G, acute renal injury can develop in the context of intercurrent infections. For example, loss of renal function in FHR5 nephropathy is associated with episodes of synpharyngitic macroscopic hematuria.^17^ The underlying C3 dysregulation in C3G patients likely results in enhanced complement-mediated renal injury after a trigger that results in renal inflammation. To model this, we induced accelerated serum nephrotoxic nephritis, an immune-complex glomerulonephritis model that involves complement and Fc receptor–mediated pathways,^18^^,^^19^ in the *Cfh*^–/–^ mice. We previously showed that *Cfh*^–/–^ mice are hypersensitive to renal injury in this setting^7^ and hypothesized that the FH_1-5_ protein, by enhancing AP regulation, could ameliorate complement-mediated renal injury in this model. Because our data indicated that the anti-P-FH_1-5_ protein was deposited in normal glomeruli, we used IgG-FH_1-5_ during accelerated serum nephrotoxic nephritis. Twenty-four hours before injection of sheep nephrotoxic serum, mice received an i.p. injection of either IgG-control (n = 7) or IgG-FH_1-5_ (n = 8). The experimental protocol is depicted in Figure 4a. Mice were culled 6 days after administration of sheep nephrotoxic serum. All 3 histologic measures of glomerular pathology (severity score, total cell number, and macrophage number) were significantly lower in the IgG-FH_1-5_ group (Figure 4b). Tubulointerstitial injury was also lower in the IgG-FH_1-5_ group (Figure 4b). Serum urea was significantly elevated in the IgG-control group, but changes in serum albumin and the urine albumin-to-creatinine ratio did not differ (Figure 4b). As expected, glomerular C3b/iC3b/C3c was significantly reduced in the IgG-FH_1-5_ group (Figure 4b), but glomerular mouse IgG (Figure 4b) and sheep IgG (data not shown) did not differ between the groups.

## Discussion

Both IgG-FH_1-5_ and anti-P-FH_1-5_ normalized C3, FB, and C5 levels in *Cfh*^–/–^ mice. This finding is consistent with the fact that the complement regulatory domains of mouse FH are located within SCR domains 1 through 5 (a C3b-binding site and the cofactor activity for the factor I–mediated cleavage of C3b).^20^ Surface recognition domains, which influence binding to heparin and endothelial cells and include a second C3b-binding site, are present within SCR domains 18 through 20 (FH_18-20_) and therefore are not present in the fusion proteins.^20^ Nevertheless, both proteins reduced glomerular iC3b/C3b/C3c, indicating that FH_18-20_ was not required in this setting. This finding is consistent with previous data showing that *Cfh*^–/–^ mice expressing a mutant FH protein consisting of SCR domains 1 to 15 (*Cfh*^–/–^.FHΔ16-20) did not develop abnormal glomerular C3 deposition.^21^^,^^22^ However, *Cfh*^–/–^.FH Δ16-20 animals were unable to regulate C3 activation along renal endothelium and developed thrombotic microangiopathy. It is possible that, owing to the lack of the surface targeting FH_18-20_ domains, administration of fusion proteins containing FH_1-5_ in FH deficiency could result in susceptibility to thrombotic microangiopathy. It is also possible that the increased avidity for C3b binding, by virtue of the dimeric structure of the fusion proteins, is sufficient to compensate for the absence of the surface targeting FH_18-20_ domains. However, surface recognition of C3b is also influenced by conformational changes in FH.^23^ A *Streptococcus pneumoniae* FH-binding protein (PspCN, which binds SCR domain 9), triggers an FH conformational change, and the FH-PspCN complex had increased C3b binding and enhanced decay-accelerating activity. The FH_20_ C3d-binding site is not exposed in FH but becomes exposed in the FH-PspCN complex. This might explain why FH_19-20_ interacts with surface C3d but FH does not.^24^^,^^25^ We speculate that our agents, through conformational changes, may efficiently interact with both fluid-phase and surface C3b. Notably, both IgG-FH_1-5_ and anti-P-FH_1-5_ protected erythrocytes from lysis in an AP-dependent hemolysis assay.

Neither fusion protein reduced glomerular C3d staining, perhaps because of the persistent nature of glomerular C3d. In experimental immune-complex nephritis, glomerular C3c resolved within 24 hours of cessation of complement activation but C3d persisted for weeks.^16^ In addition, glomerular C3d is persistent in lupus nephritis.^26^ This likely reflects its covalent interaction with glomerular surfaces. Repeated injections of human FH in *Cfh*^–/–^ mice did show a reduction in glomerular C3d over a 10-day period.^11^ It is likely that repeated dosing of IgG-FH_1-5_ might reduce glomerular C3d in this model. There was also no change in glomerular FHR staining. Little is known about the functions of the mouse FHR proteins, but they are able to interact with both C3b^27^^,^^28^ and C3d.^27^ In fact, the apparent affinity of both FHR-A and FHR-B for C3d is stronger than that of FH.^27^ We speculate that glomerular FHR proteins are likely to clear from glomeruli only when C3d has been removed. In this context, it is notable that in human C3G, factor H–related protein 5 associates with glomerular C3d.^29^

The anti-P-FH_1-5_ protein depleted free plasma properdin levels as expected, because the anti-properdin part of this fusion protein can block AP activity (due to properdin depletion) for 8 days after a single injection.^30^ However, our data showed that the efficacy of FH_1-5_ in increasing plasma C3 levels and reducing glomerular C3b/iC3b/C3c staining was comparable between the 2 fusion proteins (i.e., was independent of properdin targeting). Moreover, the abnormal glomerular properdin staining was significantly reduced after administration of either anti-P or IgG-FH_1-5_. Evidently, in contrast to C3d and FHR proteins, glomerular properdin is readily cleared after restoration of C3 regulation. Glomerular properdin changed in pattern after administration of the anti-P-FH_1-5_ protein, but this was complicated by the observation that we detected deposition of this fusion protein in both *Cfh*^–/–^ and wild-type glomeruli. This was partly due to an interaction with glomerular properdin, but the deposition in wild-type glomeruli indicated it was also in part entirely independent of glomerular complement and likely related to the physiochemical properties of the fusion protein.

Our kinetic data demonstrated novel findings with respect to the temporal changes in FB, C5, and properdin that accompany increases in C3 in *Cfh*^–/–^ mice after injection of the fusion proteins. FB levels rose rapidly after injection and returned to baseline levels at a time when both C5 and properdin levels remained elevated. Properdin levels in *Cfh*^–/–^ mice were reduced at baseline and increased to normal levels (20 μg/ml^30^) after injection of IgG-FH_1-5_ and remained at these levels for at least 14 days. A similar time course was seen for the rise in plasma C5 levels. Strikingly, IgG-FH_1-5_ was mostly absent from the circulation at 11 days, indicating that the formation of the C5 convertase in *Cfh*^–/–^ mice is slower than that of the C3 convertase (because C3 levels returned to baseline between 7 and 11 days). Plasma C5 increased after anti-P treatment and increased further with anti-P-FH_1-5_ protein. Hence, both properdin targeting and the effects of FH_1-5_ were contributing to the increased C5 levels and indicate that the C5 convertase is partially properdin dependent in *Cfh*^–/–^ mice. This finding is consistent with amelioration of C5 dysregulation in mice with combined deficiency of FH and properdin.^8^^,^^9^ Circulating properdin levels can be reduced in C3G and appear to correlate with surface C5 convertase activity in contrast to either plasma C5 or soluble C5b-9.^31^^,^^32^ Our data support an intimate link between properdin levels and C5 convertase activity and support the use of properdin levels as a biomarker of ongoing glomerular C5 activation.

Notably, the IgG-FH_1-5_ protein was still detectable in the circulation up to 11 days after a single injection. This is much longer than what we previously reported for the unconjugated mouse FH_1-5_ protein, which was removed from the circulation within 24 hours.^14^ This finding indicates that the longer plasma half-life of the IgG-FH_1-5_ protein is derived from the antibody conjugation. The plasma half-life of the IgG-FH_1-5_ protein was also longer than what we previously observed for full-length FH.^10^^,^^11^ For example, human FH was removed from the circulation by 96 hours after a single injection in *Cfh*^–/–^ mice.^11^ Combined with our data demonstrating the efficacy of the IgG-FH_1-5_ protein in complement regulation, we consider that conjugation of FH_1-5_ protein to prolong its pharmacokinetic profile has potential therapeutic utility in C3G.

In summary, we show that, despite lacking surface recognition domains, the FH_1-5_ fusion proteins were effective in reducing glomerular C3 activation and restoring plasma complement regulation in FH deficiency. There was no obvious advantage in conjugating the FH_1-5_ domains to anti-properdin, and the IgG-FH_1-5_ protein reduced renal injury in experimental nephritis. In contrast to the challenges in producing large-scale preparations of FH for therapeutic use, the large-scale production of antibody-based therapies is well established, which this makes IgG-FH_1-5_ an attractive potential therapeutic for C3G.

## Methods

### Fusion proteins

#### IgG-FH_1-5_

The first 5 mouse FH SCR domains (mFH_1-5_) linked to a non-targeting IgG1 mouse Ig (an anti-idiotypic antibody raised against a mouse monoclonal antibody).

#### Anti-P-FH_1-5_

Murine FH_1-5_ linked to mouse anti-properdin antibody (anti-P),^30^ provided to Alexion by W. Song, University of Pennsylvania).

#### Controls

Controls were isotype-matched non-targeting mouse Ig (IgG-control) and mouse anti-properdin (anti-P) (Supplementary Figure S1). Proteins were generated using pVEK vectors and Expi293 cells (Thermo Fisher Scientific, Waltham, MA), purified using protein A (MabSelect SuRe, Cytiva, Marlborough, MA).

### AP-specific hemolytic assay

Twenty percent normal mouse serum titrated from 60 nM to 0.5 nM was incubated with rabbit erythrocytes (1.5 ×10^6^ cells/ml) at 37 °C for 30 minutes. Heme release was quantified spectrophotometrically (optical density 415 nm); 100% lysis was serum with no inhibitor.

### Plasma FB and FH

Plasma was obtained after centrifugation of blood collected into tubes containing ethylenediamine tetraacetic acid (Sarstedt, Nordrhein-Westfalen, Germany). Proteins were measured by capillary electrophoresis immunoassay (WES, ProteinSimple, San Jose, CA). Antibodies used were polyclonal anti-mouse FH antibody (Alexion Pharmaceuticals, Boston, MA) and anti-mouse FB antibody (Abcam, Cambridge, UK); WES anti-rabbit detection module (ProteinSimple, San Jose, CA) was used. Chemiluminescent signals were analyzed with Compass for SW software (ProteinSimple, version 5.0.1), quantified as peak areas and normalized to the assay control.

### Mice

All mice were housed in specific pathogen-free conditions; procedures were performed according to institutional guidelines and approved by the United Kingdom Home Office. C57BL/6 wild-type mice were purchased from Jackson Laboratory (Bar Harbor, ME) and *Cfh*^–/–^ mice were generated as previously described.^7^ Mice were matched for age and sex; equimolar doses of proteins were administered via i.p. injection (1 mg for anti-properdin and IgG-control; 1.4 mg for IgG-FH_1-5_ and anti-P-FH_1-5_). Accelerated serum nephrotoxic nephritis was induced by i.v. injection of sheep nephrotoxic serum into mice pre-immunized with sheep IgG.^7^ Administration of the fusion proteins was performed 24 hours before sheep nephrotoxic serum induction (Figure 4a).

### C3, C5, and free properdin

Plasma C3 was measured by enzyme-linked immunosorbent assay.^14^ Plasma “free” properdin and C5 levels were measured using electrochemiluminescence immunoassays (Meso Scale Discovery [MSD], Meso Scale Diagnostics, Rockville, MD) with anti-properdin or a custom anti-murine C5 coated on high-binding MSD plates. Bound “free” properdin and C5 were detected with anti-properdin (from W. Song) or biotinylated monoclonal anti-C5 (Alexion) combined with streptavidin-SULFO (MSD, Meso Scale Diagnostics). The chemiluminescent signal was acquired using a SECTOR S 6000 imager (MSD, Meso Scale Diagnostics) and was analyzed with MSD Discovery Workbench software (Meso Scale Diagnostics, version 4.0.12.1). Recombinant murine properdin and C5 were used as standards.

### Renal function and histology

Hematuria and proteinurea were assessed using Hema-Combistix (Bayer, Reading, UK); plasma urea measured and kidney tissue processed as previously described.^9^ Urinary creatinine was determined on spot urine samples using the Creatinine Companion Protocol assay (#1012; Exocell, Philadelphia, PA), and spot urine/plasma albumin was measured by enzyme-linked immunosorbent assay (#E99-134; Bethyl Laboratories, Montgomery, TX). Periodic acid–Schiff stained renal sections were assessed in a blinded fashion and ranked according to the severity of glomerular and tubulointerstitial injury (0 [none], 1 [mild], 2 [moderate]). Ten glomeruli per section were assessed to determine mean glomerular cell number. Immunofluorescence staining was performed on 5-μm cryosections mounted using Vectashield medium with DAPI (Vector Laboratories, Burlingame, CA). Antibodies used were fluorescein isothiocyanate (FITC)-conjugated polyclonal goat anti-mouse C3b/C3c/iC3b (1:200; #55500; MP Biomedical, Santa Ana, CA)^9^; Fc*γ*-chain–specific FITC-conjugated polyclonal goat anti-mouse IgG (1:400; #F5387; Sigma-Aldrich, St. Louis, MO); FITC-conjugated monoclonal mouse anti-goat/sheep IgG (1:100; #F5137; Sigma-Aldrich); or FITC-conjugated goat anti-human properdin (1:50; #GAHu/PPD/FITC, Nordic Immunologic Laboratories, Copenhagen, Denmark).^9^ Biotinylated goat anti-mouse C3d (1:10; # BAF2655; R&D Systems, Minneapolis, MN) was used on biotin-blocked sections (Biotin Blocking System; Agilent Dako, Santa Clara, CA), with streptavidin AF488 secondary antibody (1:200; #S-32354; Thermo Fisher Scientific). FH was visualized in purified rat anti-mouse CD16/CD32-blocked sections (1:100; BD Biosciences, Franklin Lakes, NJ) using goat anti-human FH (1:1000; #A312; Quidel, San Diego, CA) and monoclonal anti-goat IgG-FITC secondary antibody (1:100; clone GT-34; F4891; Sigma-Aldrich). Glomerular macrophages were identified using FITC-conjugated rat anti-mouse CD68 (FA-11 clone GTX43518; GeneTex, Irvine, CA). Quantitative immunofluorescence analysis was performed using a Leica DM4B optical microscope coupled with Leica DFC700T digital camera (Leica Microsystems, Wetzlar, Germany) and Image-Pro Plus software (Media Cybernetics, Rockville, MD, version 7). Ten glomeruli were examined per section, and mean intensity was expressed in arbitrary fluorescence units.

### Statistical analysis

GraphPad Prism, version 8.0 (GraphPad, San Diego, CA) was used for statistical analysis. Repeated-measures 2-way analysis of variance with Bonferroni multiple comparisons test (factors were time and treatment) was used for the time course analysis. The Kruskal-Wallis with Dunn multiple comparisons test was used when comparing multiple groups, and Mann-Whitney testing was used for 2 groups.

## Disclosure

MCP has received consultancy fees from Alexion, ChemoCentryx, Novartis, Gyroscope, and Achillion Pharmaceuticals. HTC has received consultancy fees from Alexion, Novartis, Aurinia, and Achillion Pharmaceuticals. YZ, YW, KKJ, and SK-K are employees of Alexion Pharmaceuticals. SK is a former employee of Alexion Pharmaceuticals and is currently employed by Gemini Pharmaceuticals. All the other authors declared no competing interests.
