# Supplementary material for: Complement activity is regulated in C3 glomerulopathy by IgG–factor H fusion proteins with and without properdin targeting domains
Source: Kidney Int. 2021 Feb;99(2):396–404. doi: 10.1016/j.kint.2020.09.028 (PMC7863913; doi:10.1016/j.kint.2020.09.028)
Supplement: Supplementary File (PDF) [file mmc1.pdf]

TITLE:

Complement regulation in C3 glomerulopathy by IgG-factor H fusion proteins with and without properdin targeting domains

AUTHORS:

Alyssa C. Gilmore[1], Yuchun Zhang[2], Deborah P. Lavin[1], Suresh Katti[2], Yi Wang[2], Krista Johnson[2], SungKwon Kim[2], Matthew C. Pickering[1]

[1]Centre for Inflammatory Disease, Imperial College London, UK;
[2]Alexion Pharmaceuticals, New Haven, US

Gilmore et al Supplemental Data

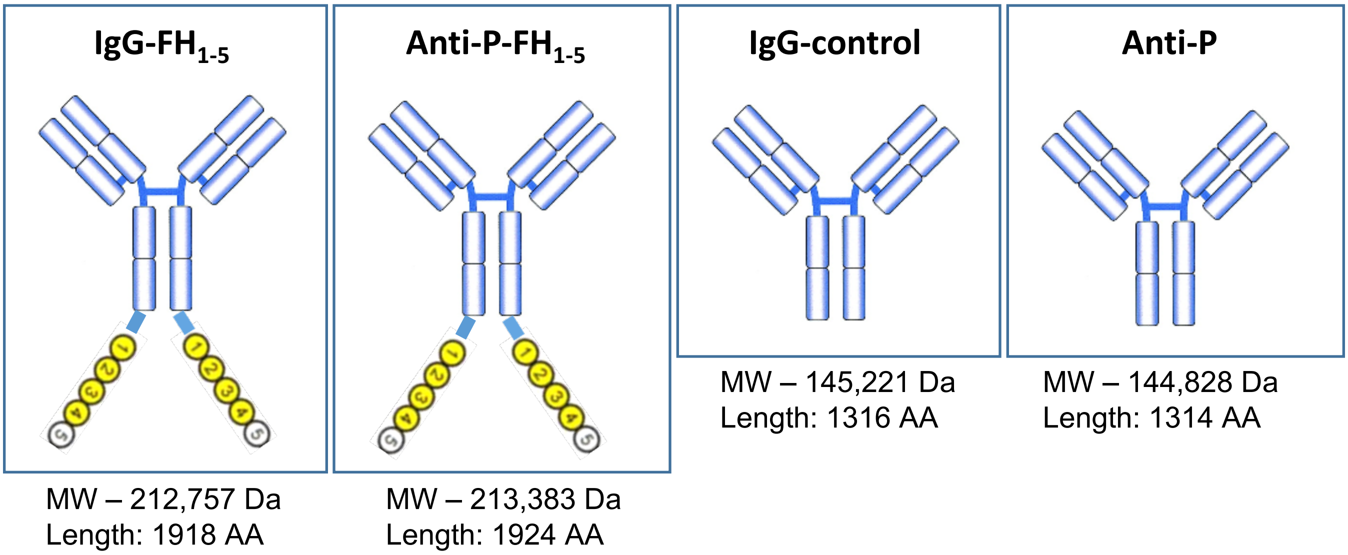

Supplemental Figure 1. Fusion proteins used in this study. IgG-FH_{1-5} contains the first five domains of mouse factor H (FH_{1-5}) linked to a non-targeting mouse immunoglobulin. Anti-P-FH_{1-5} consists of mFH_{1-5} linked to a neutralising mouse anti-mouse properdin antibody (Anti-P, see Miwa T et al., J Immunol, 190**:** 3552-3559, 2013). Controls proteins included an isotype-matched non-targeting mouse immunoglobulin (IgG-control) and the neutralising mouse anti-mouse properdin antibody (Anti-P). MW – molecular weight; AA – amino acids; Da – Daltons.

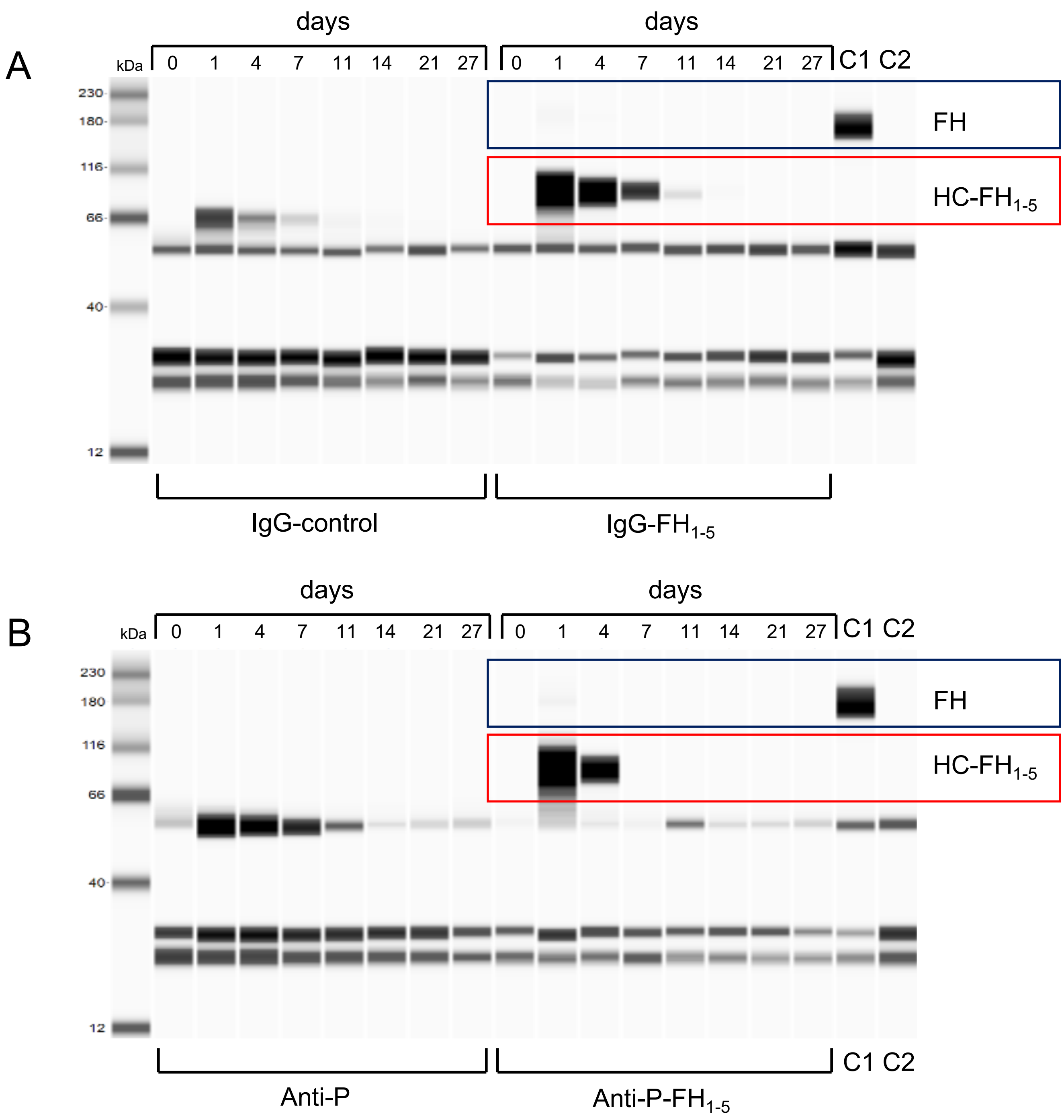

Supplemental Figure 2. WES blot analysis to detect FH_{1-5} in plasma samples from FH-deficient mice injected with either (A) IgG-FH_{1-5} or (B) Anti-P-FH_{1-5}. The 94kDa immunoglobulin heavy chain linked to mouse FH_{1-5} (HC-FH_{1-5}) is detectable up to 11 days after injection of IgG-FH_{1-5} and up to 4 days after injection of Anti-P-FH_{1-5} protein (red boxes). Controls included plasma from wild-type mice where the full length factor H protein is detected (FH, black boxes, lane C1) and non-injected FH-deficient mice, where no FH is evident (lane C2, black boxes). As expected, neither FH nor FH_{1-5} is detectable in plasma samples from mice injected with Anti-P or IgG-control.

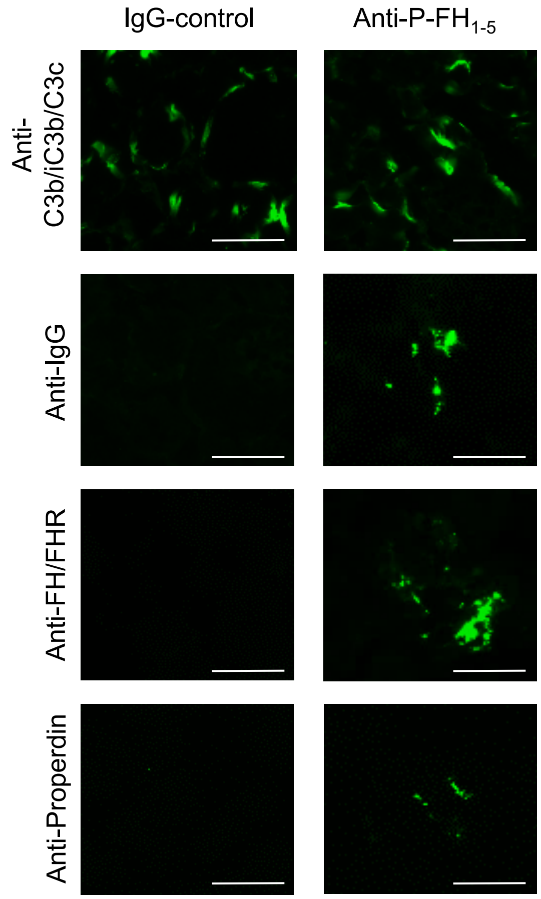

Supplemental Figure 3. Glomerular complement immunostaining 96 hours following injection of IgG-control or Anti-P-FH$_{1\text{-}5}$ in wild-type mice Representative glomerular images are shown. FH/FHR – Factor H/Factor H-related proteins. Bar 100 microns.

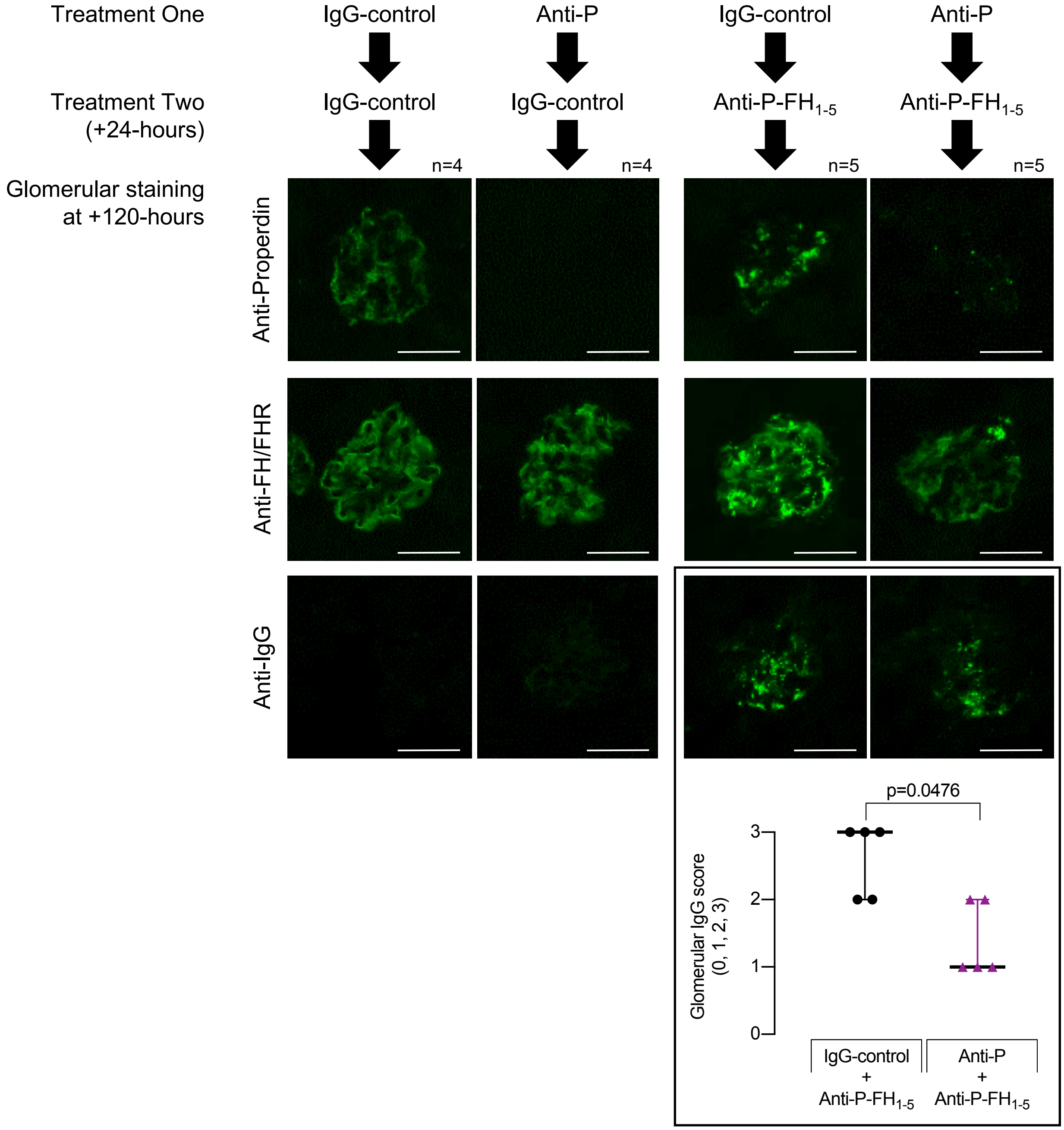

Supplemental Figure 4. The role of properdin (P) on the deposition of Anti-P-FH_{1-5} in glomeruli of FH-deficient mice. *Cfh*$^{-/-}$ mice were injected with either IgG-control or Anti-P (Treatment One) followed 24-hours later by either IgG-control or Anti-P-FH_{1-5} (Treatment Two). Animals were culled 120-hours after treatment one and glomerular staining for IgG, P and FH/FHR was assessed. Representative images are shown. Anti-P injection markedly reduced P (row one, column two) but not FH/FHR (remains unchanged) or IgG (remains absent) staining. Administration of Anti-P-FH_{1-5} 24-hours after IgG-control resulted in the appearance of granular staining with anti-IgG, anti-FH/FHR and anti-Properdin antibodies as seen following injection of Anti-P-FH_{1-5} alone (See Figure 3B). This granular staining reduced when Anti-P-FH_{1-5} injection was preceded by Anti-P injection which depletes both circulating (See Figure 1H) and glomerular (See Figure 3B) P. Glomerular staining with anti-IgG was only evident in mice injected with Anti-P-FH_{1-5} (row three, columns 3 and 4) and was significantly lower in mice that received Anti-P-FH_{1-5} preceded by anti-P. We concluded that the deposition of Anti-P-FH_{1-5} in glomeruli of *Cfh*$^{-/-}$ mice was partly dependent on P. Horizontal bars denote median values and whiskers denote interquartile range. P value derived from Mann Whitney test. Bar 100 microns.
